# How to Evaluate Health in All Policies at the Local Level: Methodological Insights Within Municipalities From the WHO French Healthy Cities Network

**DOI:** 10.34172/ijhpm.2022.6584

**Published:** 2022-07-06

**Authors:** Marion Porcherie, Marie-Florence Thomas, Frédérique Quidu, Zoé Héritage, Zoé Vaillant, Jean Simos, Stéphane Rican, Nicola Cantoreggi, Emmanuelle Faure, Anne Roué Le Gall

**Affiliations:** ^1^Department of Social Sciences, Ecole des Hautes Etudes en Santé Publique, Laboratoire Arènes URM CNRS 6051, Université Rennes 1, Rennes, France.; ^2^Ecole des Hautes Etudes en Santé Publique, Leres, Irset UMR- Inserm S 1085, Rennes, France.; ^3^Santé Publique France, Saint-Maurice, Paris, France.; ^4^LADYSS, Université Paris-Nanterre, Nanterre, France.; ^5^Institut de Santé Globale, Université de Genève, Genève, Switzerland.; ^6^Department of Health and Environment, Ecole des Hautes Etudes en Santé Publique, Laboratoire Arènes URM CNRS 6051, Université Rennes 1, Rennes, France.

**Keywords:** Policies, Local, Assessment, Quantitative, Green Spaces, Healthy City

## Abstract

**Background:** This article proposes a method for analysing the degree of maturity of Health in All Policies (HiAP) among World Health Organization-French Healthy Cities Network (WHO-FHCN) as part of the GoveRnance for Equity, EnviroNment and Health in the City (GREENH-City) project. We focused on the creation or enhancement of health-promoting environments, and more specifically, public green spaces.

**Methods:** We conducted a cross-sectional quantitative study guided by the evaluative framework of the HiAP maturity level developed by Storm et al mixed with a qualitative interpretation. A self-administered questionnaire was sent to elected officials and health department officers in the 85 member cities of the WHO-FHCN in 2017. Subsequently 58 cities were included in the analysis, which was based on a multiple correspondence analysis (MCA) and a hierarchical ascending classification (HAC).

**Results:** Thirty-two criteria among a total of 100 were identified and were used to organize the cities into 8 groups which was then reduced to three profiles among the cities: a less advanced HiAP profile, an established HiAP profile and an advanced HiAP profile. This process allows us to identify 4 dimensions that make it possible to evaluate the level of maturity of cities in the HiAP process, namely: (1) the consideration of social inequalities in health and/or health issues in the policies/actions of the sector studied, (2) occasional intersectoral collaboration, ie, one-off initiatives between the health department and others sectors, (3) the existence of joint projects, ie, common projects between two or more sectors, (4) the existence of intersectoral bodies, in this case on the theme of urban green spaces including an intersectoral committee and/or working groups.

**Conclusion:** Four dimensions which allow to the measurement of the degree of progress in implementing health-all-policies are proposed. With a view to integrating knowledge into public action, this study carried out under real conditions offers a realistic method to evaluate HiAP.

## Background

**Key Messages**

** Implications for policy makers**

- The municipalities involved in a HiAP approach will require simple criteria to assess and monitor their practice and achievement. The four dimensions identified in this article may help health sector decision-makers advocate for more consideration of the impact of the wider health determinants.
- Urban green spaces are used as an example to raise awareness amongst policy-makers of their importance for health.
- The methodology should be applied to other policy areas such as education policies or urban planning policies.

** Implications for the public**

The determinants of population health go far beyond the scope of the health-care sectors and require cross-sectoral action. Local policies can have a major impact on health and health inequalities. When applied to different sectors, such as urban green spaces, this approach can have a direct impact on health by improving the living conditions of the population, such as local infrastructures for walking, cycling or playing. By paying particular attention to the need of all, especially to vulnerable populations due to their age, physical or social conditions, this approach can create a more inclusive society.

The vast majority of the determinants of health are influenced by factors outside the health-care sector.^1,2^ Health in All Policies (HiAP) is an intersectoral approach to public policy that systematically takes into account the consequences of all political decisions in population health in formulating public policy. It seeks synergies among all different sectors and avoids negative consequences of policies on individual and population health in the interests of equity.^3-5^ The objective of this article is to propose, through a cross-sectional implementation study, a method for analyzing the degree of HiAP maturity of cities based on the framework proposed by Storm et al.^6^ Our study has a particular focus on health-promoting environments, specifically public urban green spaces.

 This study was conducted in partnership with the World Health Organization-French Healthy Cities Network (WHO-FHCN) in the framework of the GREENH-City project.^7^ The city members of this network are committed to adopting a HiAP approach as a mode of governance.^8^ Different conceptions of HiAP implies different operational implementation, as well as its level of deployment. Studying the implementation of HiAP is crucial to ensuring all determinants of health are taking into account when developing public policy.

###  Conceptions and Implementation of a HiAP Approach

 The implementation of HiAP, whether at the national or local level, depends on the extent to which health issues are central, to collaborative activities between services.^9-11^ To varying degrees, many countries have taken up this approach and integrated it into their health governance systems. Literature has been published during the last ten years on the theoretical conceptualization of the HiAP approach and models to explain its functioning,^12,13^ facilitating factors and its capacity to be sustained over time.^14^ Recent publications^15,16^ show that most of the scientific production is concentrated at the national or regional level, or at least at state or federal decision-making levels. In some countries, studies are beginning to look at the implementation of these top-down strategies at the local level, such as Norway,^17^ where the principle of HiAP is integrated into the governmental system, or the Netherlands.^18^

###  Local Level: Preferred Level to Operationalize the HiAP Approach

 Local policies, particularly those defined at the city level, are likely to have a greater impact on health equity.^19^ At this level, there is greater proximity between decision-makers and the population, and between decision-makers from different sectors, a proximity likely to promote intersectoral collaboration and therefore action on living environments.^20^ In theory, the local level is the best place to operationalize HiAP and to promote health and equity.^21,22^ Although there are still few published studies, local-level experiences in implementing a HiAP approach have received increasing attention in the last two years.^23,24^ Factors supportive of HiAP are known. These includes a shared vision of HiAP among the actors involved, funding to support the approach, ownership and accountability, local leadership and a dedicated team, health impact assessments, and the existence of local health indicators and process indicators.^25^ However, objectifying effective implementation of the HiAP approach remains a challenge and requires innovative evaluation approaches.

###  Challenges of Evaluating the Implementation of HiAP

 Process and outcome evaluations of HiAP are complex.^13,26^ Until now, evaluations have used a realist approach, which focuses on characterizing the mechanisms that facilitate the meeting of different sectors (health, urban planning, education, social, etc), according to the context of their intervention and the actors involved.^27,28^ Qualitative description has also been used to report the results in terms of the success of their implementation.^12^ Van Vliet et al^23^ point out that current research mainly produces recommendations or proposes narrative evidence of the enlistment of different policy sectors around a given health issue, without giving the keys to their success or failure. The aim of most evaluations is to assess the conditions that facilitate the implementation of cooperative approaches rather than the actual degree of implementation of the approaches.^6^ At the local level, there is little literature proposing this degree of implementation evaluation. Publications focus only on specific aspects of the HiAP approach, such as its inclusion in urban planning policies that are favourable to health,^29^ or testify to its deployment by evaluating the integration of decision-makers’ knowledge on equity.^30^ In our study, we wanted to estimate the level of concrete implementation of HiAP amongst healthy cities members, following the adoption of the Copenhagen Consensus.^31^ This reaffirms that, in addition to promoting health and well-being, cities must work to create urban environments that contribute to equity and prosperity for their inhabitants.^32,33^ This type of a HiAP-based public action is prevalent in the engagement of healthy cities.^34^ Although by joining the network, cities commit themselves to respecting its ethical principles, the implementation of HiAP is no homogeneous. There is a need to establish a practical approach to assess the level of HiAP among stake-holders such as municipalities. This is particularly true in the case of France because, unlike some other countries, there is no specific strategy at the national level proposing an operational implementation of HiAP.^35^

 Based on the Storm’s framework, our study aimed to propose a new method for analysing the degree of maturity of the HiAP approach. It measured the WHO network of healthy cities’ commitment to HiAP and to consider where the issue of urban green spaces is situated among their policies.

 Urban green spaces are one of the environmental determinants of health for populations living in cities.^36^ Maximizing the positive effects of urban green spaces depends on their design, accessibility, and nature, and minimizing their potential negative effects (such as the presence of allergenic plants, pests, lack of maintenance, etc^37^). The benefits of urban green spaces are now well documented in the scientific literature, but some public authorities still ignore these multiple advantages in their urban plans.^38,39^

## Methods

 Our quantitative study measured HiAP among 85 municipalities from the WHO-FHCN.

###  Storm et al HiAP Framework (2014) 

 The conceptual framework developed by Storm et al along with two other sources^9,38^ were the basis for the design of the collection tool (questionnaire) and also the analytical framework for analysing the degree of maturity of the HiAP approach among municipalities who replied.

 The framework proposed by Storm at al,^6^ draws on management science, to describe the five levels of HiAP maturity – recognized, considered, implemented, integrated, institutionalized. It is based on the description of 14 characteristics related to the consideration of social inequalities in health issues (see Table 1). They aims to compare the level of collaboration between services and the level of consideration of social inequalities in health. Detailed explanations of each of the 14 characteristics are available in the original article. As we wanted to test the consideration of social inequalities in health, of health more generally and of green spaces interventions, we adapted the Storm framework by expanding the scope of some questionnaire items.

**Table 1:** Levels of Maturity of the HiAP Approach at the Local Level and Their Main Characteristics According to Storm et al^6^

**Maturity Degrees **	**Characteristics **
The HiAP approach is recognized	1. Importance of HiAP recognized to reduce health inequalities 2. Visible which activities of sectors contribute to (determinants of) health inequalities
The HiAP approach is considered	3. HiAP described in policy documents 4. Collaboration with sectors present (project-based) 5. Collaboration on health inequalities is started 6. Activities of sectors contribute to determinants of health inequalities
The HiAP approach is implemented	7. Concrete collaboration agreements 8. Structural consultations forms present 9. Key person HiAP is present (role is clear) 10. Working from sectors on health inequalities (policy basis)
The HiAP approach is integrated	11. Broad, shared vision on HiAP (political and strategic) 12. HiAP results visible (both content and process)
The HiAP approach is institutionalized	13. Political and administrative anchoring of the HiAP 14. Continuous improvement of integral processes and results on the basis of the achieved results

Abbreviation: HiAP, Health in All Policies.

 We used the different degrees of maturity described by Storm et al to analyse the results and measure the levels of collaboration between municipal services, particularly between health and green spaces as it is described in Data Analysissection.

###  Material and Data Collection Method

**Figure 1:**
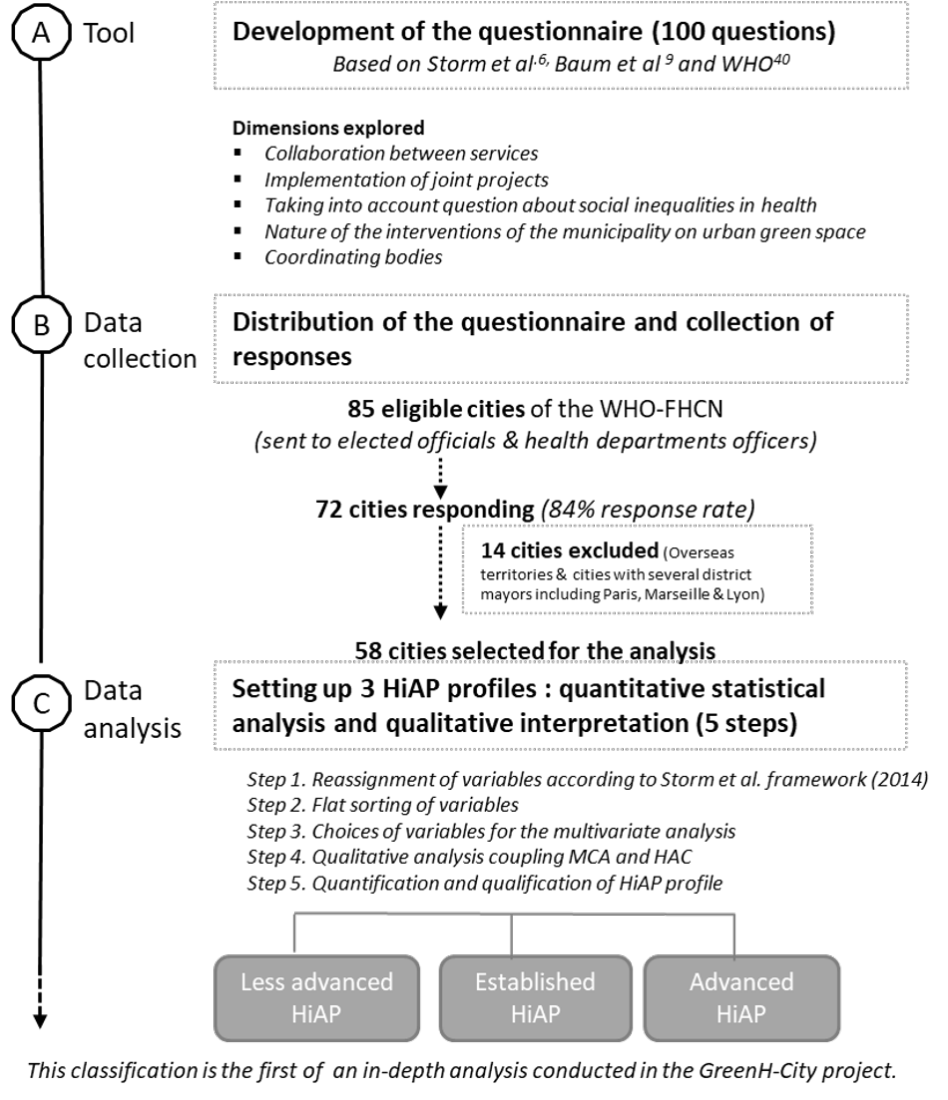
Main Steps of the HiAP Maturity Measurement Method Developed With Municipalities Members of the WHO French Healthy City Network (2017, France). Abbreviations: HiAP, Health in All Policies; MCA, multiple correspondence analysis; HAC, hierarchical ascending classification; WHO-FHCN, World Health Organization-French Healthy Cities Network.

 The different steps of the methodology to determine HiAP profiles is summarized in Figure 1.

####  Respondents: The Cities of the WHO French Healthy Cities Network 

 All 85 cities that were members of the WHO-FHCN at the time of the study were surveyed. Data were collected by self-administered questionnaire from these cities between July 2017 and August 2017, targeting only elected officials and officers from departments in charge of health or related issues. These people were targeted as they are expected to support HiAP in accordance with the Phase VII of WHO European Healthy Cities Network.^31^ Respondents were asked to complete only one questionnaire per city, giving priority to consultation between elected officials and officers. Understanding the ways in which different sectors collaborate helps to understand the varying degrees of focus on health issues. In this area, desirability bias can be strong. We can rely on the veracity of negative responses about joint projects, which means that when they say they are not collaborating, we can believe that they are not.

####  Criteria for Excluding/Eligibility of Cities for Inclusion in the Analysis 

 Among the cities that responded to the questionnaires, we chose to exclude cities with a specific administrative organization, such as the cities of Paris, Lyon and Marseille, which have several district mayors. We also excluded cities from overseas territories and departments, as well as territorial groupings of municipalities for their specific political organization that differs from a municipality (see Figure 1).

####  Collection Tools: The Questionnaire 

 The questionnaire was constructed to determine (1) the maturity of HiAP in particular, (2) and how health and equity issues were integrated into their green spaces policies.

 More specifically, we sought to understand:

1. Whether health was placed at the heart of each municipal sector of activity (for example, social sector, children’s services, urban planning, etc) or whether health issues are only of interest to the health sector ie, the type of strategies implemented based on Baum et al.^9^
2. Whether or no municipalities have the conditions to implement HiAP using the Storm et al framework.^6^
3. Whether municipalities using health-promoting components of green spaces interventions as defined in WHO expert reports^40^ as part of HiAP considerations.

The questionnaire contained 100 questions, including both closed and open-ended questions. The questionnaire is available in French in Supplementary file 1.

 During the analysis, the open-ended questions were recoded to transform them into quantitative variables (see Table S3 in Supplementary file 2). We performed a qualitative content analysis of each open-ended-question to determine the nature and number of quantitative variable classes to recode variable when similar responses were given. For example, when asked “are you a member of a social service body or committee? if so, please detail which one,” we found three different themes that grouped the most frequent responses: social service center, disability committee or both. A variable was then created and coded into 4 modalities covering the three types of responses, plus one modality coding all other responses (see Table S3 in Supplementary file 2).

###  Data Analysis 

 In order to create the cities HiAP profiles from their questionnaire responses, we carried out a quantitative statistical analysis and a qualitative interpretation of their results which took place in 5 steps:

####  Step 1. Reassignment of Variables to a Category in the Storm et al Framework 

 Each variable of the questionnaire was linked to a HiAP maturity degree according to Table 1. In addition, each of these variables was classified as explanatory of a formalized HiAP approach (ie, based on actions – project sharing, interdepartmental committees, etc) or an informal one (no project sharing but only interpersonal exchanges) (see Table S1 in Supplementary file 2).

####  Step 2. Initial Data Organisation

 During this step, we recoded scattered qualitative variables as explained in Storm et al HiAP Framework section (see Supplementary file 2). Also we eliminated variables which had a lot of missing replies.

####  Step 3. Choice of Variables for the Multivariate Analysis 

 As the sample was quite small (n = 58) and the number of variables large, we selected only those variables which specifically explained the degree of the HiAP maturity.

 Based on the classification of variables determined in step 1, we then selected 32 variables that best characterize HiAP maturity (see Supplementary file 2). Here we chose to eliminate from the analysis the variables related to an informal HiAP approach (for example, when they only related to interpersonal relationships). We kept the variables directly related to collaborations between services, implementation of joint projects, taking into account health and social inequalities of health, the existence of green spaces interventions (related to health, equity or environment) and the existence of a health coordination committee inside municipality.

####  Step 4. Determination of the Profiles Via a Quantitative Analysis Coupling an MCA and a HAC Using a Taxonomy

 In order to create city profiles reflecting their HiAP, we implemented a multiple correspondence analysis (MCA) coupled with a hierarchical ascending classification (HAC).^41^ The MCA that we carried out with SAS© enabled us to identify the most discriminating variables between the cities with regard to their implementation of the HiAP approach and to eliminate those that were redundant (because they are correlated with each other). These variables were then used in the HAC to build the profiles.

 We opted for a HAC because we did not want to fix in advance the number of homogeneous groups of cities.

####  Step 5. Quantification and Qualification of HiAP Profiles 

 The statistical analyses created homogeneous groups of cities, ie, those with similar profiles.

 In order to interpret these profiles, we conducted a qualitative analysis consisting of the analyse of each variables (ie, answers of the questionnaire). The aim was first, to understand which characteristics were used to group cities into similar classes. Secondly, to analyse their degree of progress in the implementation of a HiAP approach according to the Storm categories.

## Results

###  Identification of Eight Classes of Cities

**Figure 2:**
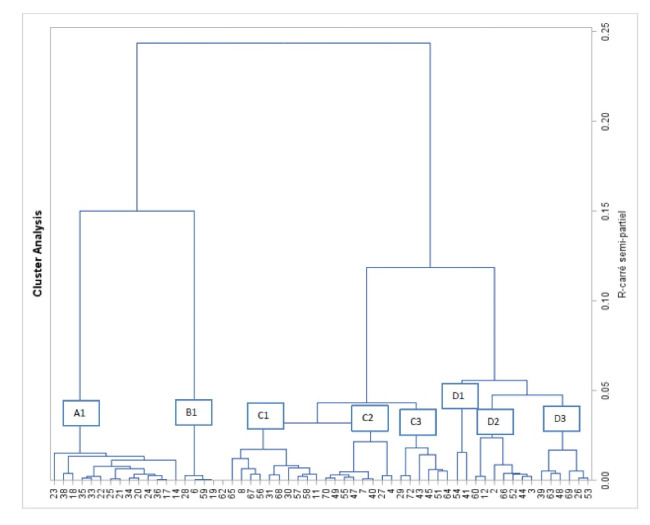
Dendrogram Resulting of the Hierarchical Ascending Classification Analysis and Identification of Classes by Profile (A1, B1, C2, C2, C3, D1, D2, D3).

 Out of 85 eligible cities, 72 cities responded to the questionnaires (84% response rate). According to the inclusion criteria, 58 responding cities were included in the analysis (see Figure 1, Figure 2 and Table 2).

**Table 2:** Most Frequent Answers of the 32 Variables Used in the Analysis (n = 58 Cities) and Classified by Themes

**Classes**	**Criterion**		**City Classes**							
			**A1**	**B1**	**C1**	**C2**	**C3**	**D1**	**D2**	**D3**
Collaboration between services										
*(Part II of the questionnaire)*	Social/handicap		NA	NA	No	No	Yes+/no	Yes++	Yes/no	Yes + yes other
	Urban planning		NA	NA	No	No	No	No	No-/yes	Yes, ++ Yes, ++ Yes, ++ Yes, ++ Yes, ++ Yes, ++ Yes
	Childhood		NA	NA	Yes	No	No/yes	No/yes	Yes + No	Formal yes + other
	Urban planning		NA	NA	No	No	No	No	No-/yes	Yes ++ No
	Sport		NA	NA	No	No++	No	Yes++	Yes other/yes	Yes ++ No
	Habitat		NA	NA	No++	No+/yes,dk	No+/yes	No	Yes+/no	No/yes/dk
	Nutrition		NA	NA	No+	No++	No+	Yes/no	No/yes	None/yes,dk
	Sustainable develop		MISSING FRQS							
	Green spaces		NA	NA	No++	No+/dk	No++	No/dk	No+/yes	dk/NA/NA
		**Total **	NA	NA	No	No	No/yes	Yes/no	Yes other	Yes++ formal
Implementation of joint projects										
*(Part II of the questionnaire)*	Social/handicap		Yes++	NA+/yes	Yes+/NA/no	Yes+/NA/no	Yes+/no	Yes++	Yes++	Yes++
	Urban planning		No+/yes	NA+	Yes+	No+	No+	Yes = NA	Yes++	Yes+
	Childhood		Yes++	NA++	Yes++	Yes++	Yes+/no	Yes++	Yes++	Yes++
	Sport		Yes + No	NA+	Yes++	Yes = no	Yes+/no	Yes++	Yes++/no	Yes++/dk
	Habitat		Yes+/no	NA++	No+/yes/dk	No+/yes	No+/yes	No++	Yes+/no=NA	Yes+/no
	Nutrition		MISSING FRQS							
	Sustainable develop		Yes=NA/no	NA++	Yes	No = yes	No/yes	Yes++	Yes+	Yes+
	Green spaces		Yes=no	NA++	Yes	No+/yes	No/yes	Yes++	Yes+/no = NA	Yes+/NA/no
		**Total **	Yes-	NA	Yes	No/yes	No/yes	Yes++	Yes++	Yes++
Taking into account questions about SIH/Health		*HI*								
*(Part II of the questionnaire)*	Social/handicap		2 +	NA++	2	2	sih+/2	2++	2++	2++
	Urban planning		dk/2 = sant = sih/pas	NA+	Health/health	None = dk	dk/2 = health = none = NA	Health=2	2+	2++
	Childhood		2+/health = sih/NA	NA++	2+/health/sih	2+/health	3+/health=nohing	2++	2+/health	
	Sport		2+/health	NA++	Health+/2/sih = none	2+/dk = health	Health+/none = 2	2++	2 = health/sih	2++
	Habitat		dk/NA/health = 2	NA	Health=2	dk/2/sih = health	None/2	dk++	2+/no dir/2	2++
	Nutrition		Health+/2/dk = no dir	NA++	Health+/2	Health+/no dir = sih = 2	None = health = 2	Health=dk	2+/health	2++
	Sustainable develop		2/no dir/health/dk	NA++	Health = 2 = no dir	2/sih=health	None+/no dir = health	Health = 2	2+/dk = health = no dir	2++
	Green spaces		Health/sih	NA++	Health/sih	dk+/health/2	None++	Health++	2++/no dir	2 = health = no dir
		**Total **	2 or health	NA	Health	dk or 2	sih or none	2 or health	2+	2++
Nature of the interventions of the municipality on urban green spaces	Environmental themes		NA	NA	None+/4.6/1.3	None++/1,3 = 4,6	None/1.3		4,6/1,3	1.3 +/no,NA
*(Part III of the questionnaire)*	Theme modes		NA++	NA++	1,2/4 = 1/3 = NA	None = 3/2	1/2/3	3=4	4++	2 = 3
	Equity theme		NA++	NA++	None/NA/2/1	1/2,3	None+/1 = 2	1 = 2	4++	2,3++
	Health themes		NA++	NA++	0,1+/1,2	0,1++	NA+/1,1 = 2 = 5	1,2 = 5,6	1,2 = 2,3 = 5,6/1	3,4 = NA+/0 = 5
Coordinating bodies	Environment Working Group		NA++	NA++	No+/NA	No++	No+/yes no env	dk++	No+/yes env/NA	Yes no approx+/no
*(Part IV of the questionnaire)*	Committee		No+/yes/dk/NA	NA++	No++	No++	No++/yes	Yes = dk	No+/ yes = dk = NA	Yes = no
	Working Group		Yes = no/NA	NA++	No+/NA/dk	No++	No+/yes	dk++	No+/yes = NA	Yes+/no/NA

Answers are given in descending order of importance in terms of number of items. NA = no answered, + = majority presence, ‘=’ = number of identical items between the different answers, MISSING FRQS = missing frequency (item no answered), dk = don’t know, SIH/sih= social inequalities in health, no dir = no service in this field. The numbers (0, 1, 2...6) represent the number of items answered to this question (multimodal), 6 being the highest score.

 The HAC based on 32 variables produced the dendrogram shown in Figure 2. The variables provided led us to determine 8 different classes comprising cities with similar profiles (A1, B1, C1, C2, C3, D1, D2, D3).

 Each city classes replies are listed in Table 2. It summarizes the most frequent responses in number of items for each of the 32 variables kept for the analysis. In addition to this quantitative approach, we performed a qualitative analysis of the answers in order to define the HiAP profile. To help us in our interpretation, we used our classification of variables by degree of the HiAP maturity. For each question, we assumed that the more positive the reply, the higher the degree of the HIAP maturity. The profiles of each group were compared and some were combined together. These qualitative analyses of the profiles ultimately resulted in three profile types, which are presented in Table 2.

###  Three Maturity Profiles of Health in All Policies 

 We identified three standard profiles depending on the degrees of maturity in the HiAP approach from less to more advanced. Number of cities per MCA groups and per profile can be found in Table 3.

**Table 3:** Number of Cities Per MCA Classification Criteria and the HiAP Profile

**MCA Criteria**	**A1**	**B1**	**C1**	**C2**	**C3**	**D1**	**D2**	**D3**
Number of cities	14	5	10	8	6	2	7	6
HiAP profile	Less advanced		Established				Advanced	
Number of cities and (% of total)	19 (33%)		26 (45%)				13 (22%)	

Abbreviations: HiAP, Health in All Policies; MCA, multiple correspondence analysis.

####  The Less Advanced HiAP Profile 

 This profile includes two classes (A1 n = 14 and B1 n = 5) for which the questionnaire items were partially completed. Intersectoral collaborations and joint projects, when they exist, tend to be with sectors who have traditionally cooperated with the health sector, ie, social/disability services and the early childhood service, sports and housing departments. Respondents indicate that these four sectors also have a focus on health issues and social inequalities in health.

####  Established HiAP Profile 

 This profile includes three classes (C1 n = 10, C2 n = 8 and C3 n = 6) that present a low response rate for items related to collaborations. They declare less joint projects with other sectors than the other groups of cities. We have considered that a low response rate indicates less involvement with other services. Joint projects, when they exist, are rather carried out with sectors traditionally oriented towards the health sector, ie, social/disability and the early childhood and sports departments. For class C1, they also existed with the sustainable development and green spaces sectors. Health issues seem to be dealt with at a minimum by the different sectors and issues of social inequalities in health. The two cities of class D1 are close to classes D2 and D3, but it was included in this profile as information was missing concerning the existence of environmental working groups and intersectoral bodies.

####  The Advanced HiAP Profile 

 This profile groups together two classes (D2 n = 7 and D3 n = 6) that are distinguished by the frequency of their positive responses indicating formal intersectoral collaboration (a committee, for example) and also in the implementation of joint projects with all sectors, including green spaces. Unlike class D1, classes D2 and D3 also mention the existence of environmental working groups and inter-sectoral bodies, in a majority way for D3 and less so but still present (2nd most frequent response) for the D2 class.

 To sum up, 19 of the 58 (33%) cities have characteristics associated with a less advanced HiAP profile. In this profile, intersectoral action is rare but health and equity concerns are present. The established HiAP profile group includes 26 cities (45%). These cities are generally involved in cross-sectoral action with more traditionally health-friendly sectors, but have little involvement in environmental issues including green spaces, without any inter-sectoral bodies, with the exception the cities belonging to the D1 class. The advanced HiAP profile includes fewer cities (only 13, 22%), but these are distinguished by a strong commitment to environmental issues including green spaces which can be illustrated by health-environment bodies and specific working groups.

## Discussion

###  Contribution of the Method to Defining the Criteria for Measuring the HiAP Maturity Degree 

 There is no one established tool kit on how to implement health approach in all policies, nor is there a consensus on how to evaluate them. It is a complex and evolving concept that differs according to the socio-political contexts of the countries that implement them. We propose here a way of looking at the concrete implementation of HiAP based on the level of collaboration between health services and green spaces services in France.

Table 2 shows less Yes++ and less 2++ replies for green spaces sectors compared to other sectors such as social/handicap, urban planning or sport. The HiAP approach is more frequently found with sectors such as the social sector or childhood and education services where collaborations, sometimes long-standing, are more easily established than with green spaces and/or environmental sectors. We identified some cities with an advanced profile, where an intersectoral approach already existed between the city’s green spaces department and the health department, or at least, bodies are in place, which promote collaboration. Green spaces development can be a cross-cutting issue for many municipal sectors such as with:

- the education sector, for example the greening of schoolyards, good playground facilities,
- the urban development sector, ie, the greening of bicycle paths or the creation of new green spaces,
- or transportation, to ensure equitable access to green spaces via public transport or sustainable mobility modes (cycling, walking, etc).

Our study focuses on urban green spaces cooperation with the health sector policies but a similar approach could be taken to assess other policy collaboration. Finally, for the qualitative interpretation of the results for each variable to define the HiAP profiles, we did not only use variables linked to green spaces interventions. The most discriminating variables were related to the following 4 dimensions:

- The consideration of social inequalities in health and/or health issues in the policies/actions of the sector studied,
- Occasional intersectoral collaboration, ie, one-off initiatives between the health sector and others,
- The existence of joint projects, ie, common projects between two or more sectors,
- The existence of intersectoral bodies, in this case on the theme of urban green spaces including an intersectoral committee and/or working groups. The latter could also concern other sectors.

These 4 dimensions could be used as an assessment tool as they indicate the gradual progression of HiAP maturity (see Table 4).

**Table 4:** Health in All Policies Assessment Tool

**Dimensions**	**Yes/No**	**HiAP Profile**
Are social inequalities in health and/or health issues considered in the policies/actions of the sector studied?	Yes/no	HiAP is recognized
Does occasional intersectoral collaboration, ie, one-off initiatives exist between the health sector and others?	Yes/no	HiAP is taken into consideration
Does joint projects, ie, common projects between two or more sectors exist?	Yes/no	HiAP is implemented
Are intersectoral bodies and/or working groups settled?	Yes/no	HiAP is institutionalized

Abbreviation: HiAP, Health in All Policies.

 The first step would be for cities to take both health and also social inequalities of health dimensions into account (level 1 of Storm classification, ie, HiAP is recognized). The second step, would be to actively promote intersectoral collaboration (Storm level 2 ie, HiAP is taken into consideration), the third step would be to implemented joint projects (Storm level 3 and 4 ie, HiAP is implemented). The last step would be run an intersectoral body or permanent working groups (Storm level 5 ie, HiAP is institutionalized). The creation of these dimensions was not planned but it contributes to Van Vliet-Brown et al^23^ call for the production of indicators that can be used by municipalities to measure their success in implementing HiAP. We provide four simple dimensions produced from real-life conditions which reflect actual practices. For example, these dimensions could be used as binary indicators (presence/absence) and be proposed as a simple dashboard to decision-to monitor the HiAP maturity degree in their city. In comparison with the current methodologies for assessing the HiAP approach, our methodology adds a practical tool of simple indicators that are easy to used by decision-makers.

###  Internal and External Validity of the Characterization of the HiAP Approach

 To test the internal validityof our methodology, we plan to compare the HiAP profiles based on the questionnaire analysis with a qualitative analysis based on in-depth interviews with elected officials and officers of a small sample of municipalities. To be discriminating by social inequalities in health, we selected cities that have a level of socio-economic inequality higher than the average for the sample of respondents. The results of the in-depth analysis are on-going and will be published soon. However, the first results ofon-site interviews seem to confirm the profiles described in this paper. This in-depth study will also help tocontextualize the results observed and provide understanding useful to better predict the transferability of the results such as the local organizational, social, historical, economic, population or cultural contexts of the municipalities as highlighted by Guglielmin et al.^24^ The results of the context assessment through our in-depth qualitative study will validate the accuracy of our methodology for evaluating the HiAP approach.

 Concerning the external validity of our methodology, firstly, we did not use the specific questions related to green spaces in order to establish the cities profiles. The 4 dimensions could be applicable to all domains and policy sectors of the municipality who would like to assess their HiAP achievement. Another way to test this external validity would be to monitor the potential effect on population health and well-being among municipalities presenting different levels of HiAP maturity. Since HiAP impacts a large number of health determinants and health equity through different policies, we could track a series of indicators related to different key health determinants such as air quality, walkability of the municipality, cycling infrastructure, etc. Finally, this study could also be extended internationally, allowing us to test the transferability of our evaluation criteria to other contexts.

###  Limitations of the Study 

 Many of the questions in our study were concerned with social inequalities in health, the answers to which are likely to include a social desirability bias,^41^ as this issue should be a major concern of the respondents as they are part of the healthy cities movement.^28^

 In comparison with Storm et al, the response rate was particularly high compared to the returns that FHCN normally records (84% of respondents our study vs 32% in Storm’s study). It mitigated the respondent selection bias where, generally, the most concerned individuals respond. Finally, asking health services about other services may have led to recall bias.

 Also very different cities responded to the questionnaire. The cities respondents had populations ranging from less than 30 000 to more than 200 000. Overall, the advanced HiAP group contained more large cities than the other groups. It can be assumed that small cities with few resources cannot set up intersectoral collaborations in the same way than a large city with several departments. The criterion of the size of the city, and also that of the way services are organised, should be explored further.

 In addition, our results may be less consistent for the less advanced HiAP profiles because for these profiles, some responses were missing. We assumed that they responded partially because they were not fully engaged in the HiAP approach. However, the questionnaire may have taken too long to complete. For these cities, it would be interesting to propose a short set of indicators to see if they fit the profile determined by our questionnaire. Finally, we had initially asked the green spaces departments to complete a questionnaire to establish their point of view of collaboration with the health departments and their level of awareness of social inequalities in health. Unfortunately as lack of replies meant, it could not be analysed.

## Conclusion

 This article proposes a method for analyzing the degree of HiAP maturity at the local level is illustrated through considering specifically the cooperation between health and green spaces policies. It is based on a statistical classification method using MCA, enriched by a qualitative analysis. The results of this analysis allowed us to identify three main profiles among the cities studied according to their level of maturity in HiAP.

 Storm et al proposed a framework containing fourteen indicators aimed at taking into account social inequalities in health. Inspired by this framework, our results also enabled us to propose four dimensions which measure the degree of HiAP progress, (1) awareness of social inequalities in health and/or health issues by the sector under consideration, (2) level of collaboration between sectors, (3) existence of joint projects and finally (4) creation of effective intersectoral bodies and their outputs.

 Our study gives a realistic vision of the HiAP approach implemented among municipalities who are members of the French WHO Healthy city network.

 The findings are in line with the conclusions of the evaluation of phase V of the WHO European Healthy Cities Network using a realistic review.^42^ It identified three main types of intersectoral actions carried out in cities, namely intersectoral governance, intersectoral action and intersectoral policies. The indicators we propose cover similar fields.

## Acknowledgements

 The authors acknowledge Dr. Jeanine Pommier and Linda Cambon for their support at the beginning of the study and the Réseau français des villes-santé which helped to disseminate the questionnaire and produce the results, especially Clément Bader.

## Ethical issues

 The GREENH-City project received prior ethics approval from Université Paris Descartes (Comité d’Evaluation Ethique pour les Recherches en Santé - N°2017-36). The project has been carried out with full respect of current relevant legislation (eg, the Charter of Fundamental Rights of the EU) and international conventions (eg, Helsinki Declaration).

## Competing interests

 Authors declare that they have no competing interests.

## Authors’ contributions

 MP wrote the article, designed the questionnaire and analysed the qualitative data, made the interpretation and drawn the results. FQ made the statistical anlaysis and drafted the paper. ARLG and MFT revised the paper. JS, NC, ZV, EF, ZH, and SR revised the paper. All the authors approved the draft.

## Funding

 The GREENH-City projet is funded by the National institute for Cancer, France (RI 2017-03).

## Supplementary files

Supplementary file 1. Questionnaire HiAP French.

Supplementary file 2. Variables Classification for Analysis.
