# Supplementary material for: How to Evaluate Health in All Policies at the Local Level: Methodological Insights Within Municipalities From the WHO French Healthy Cities Network
Source: Int J Health Policy Manag. 2022 Jul 6;11(12):3060–70. doi: 10.34172/ijhpm.2022.6584 (PMC10105196; doi:10.34172/ijhpm.2022.6584)
Supplement: Supplementary file 1 — Questionnaire HiAP French. [file ijhpm-11-3060-s001.pdf]

Marion Porcherie, EHESP, Arènes UMR 6051 Université Rennes 1, France : marion.porcherie@ehesp.Fr

Questionnaire GREENH-City

Ce questionnaire destiné aux membres du Réseau Villes-Santé, adressé dans le cadre du projet **GREENH-City**, doit être rempli par **un(e) élue(e) à la santé ou un(e) technicien(ne), ou les deux ensemble. Un seul questionnaire doit être rempli et renvoyé par chaque Ville-Santé**. Son temps de remplissage est estimé à 30 min.

De vos réponses dépend la bonne réussite du projet! Les informations que vous allez nous fournir seront essentielles pour cette recherche et pour les actions futures du Réseau. Merci par avance de consacrer un peu de temps pour y répondre!

Vos réponses sont automatiquement enregistrées à chaque changement de page. A tout moment, vous pourrez reprendre le questionnaire en cliquant sur le lien qui vous a été adressé.

Partie 1/5 - Questions générales

1. Caractéristiques générales

a. Nom de votre ville :

b. Nom(s) Prénom(s), de la/des personne(s) qui rempli(ssen)t le questionnaire :

c. Courriel :

d. Êtes-vous :

- Technicien(ne)
- Elu(e)
- Elu(e) et technicien(ne) ensemble

Quel est votre service/direction de rattachement ?

Quel est l'intitulé de votre délégation ?

Quel(s) est/sont votre(vos) service(s)/direction(s) de rattachement?

d. Êtes-vous en poste...

- depuis moins de 3 ans
- entre 3 ans et 5 ans
- depuis plus de 5 ans ?

2. Le service santé dans votre ville

2. Dans votre ville, un service santé :

- Est explicitement identifié
- Est regroupé avec un ou d'autres services
- Ce service n'existe pas
- Autre réponse (précisez)

Autre réponse (précisez) :

Le(s)quel(s) ?

Partie 2/5 - Prise en compte de la santé dans les différents services / directions de la ville

1. La santé dans le service / direction ACTION SOCIALE (et/ou HANDICAP)

a. Selon vous, dans la politique qu'il déploie, est ce que le ***service/direction action sociale - handicap*** *prend en compte :*

- ☐ Les questions de santé
- ☐ Les inégalités sociales et territoriales de santé
- ☐ Aucune de ces questions
- ☐ Ce service / cette direction n'existe pas
- ☐ Ne sais pas

(plusieurs réponses possibles)

b. Menez-vous des projets communs ou des actions communes avec ce service/direction?

- ○ Oui
- ○ Non
- ○ Ne sais pas

Si oui, veuillez citer ***un exemple*** *phare d'action commune :*

c. Siégez-vous (ou votre service) à ***un comité ou instance du service/direction action sociale - handicap*** *(type conseil d'administration du CCAS, commission d'accessibilité des personnes en situation de handicap, commission locale d'insertion sociale et professionnelle (CLI), etc.)?*

- ○ Oui
- ○ Non
- ○ Ne sais pas

Si oui, le(s)quel(s)?

Si non, pourquoi?

- ○ Ce type de comité/instance n'existe pas dans ma ville
- ○ Ce type de comité n'existe pas mais d'autres modes d'échanges informels existent
- ○ Ce type de comité existe mais je n'y vais pas
- ○ Autre raison (précisez)

Autre raison (précisez) :

Quels autres modes d'échanges informels existent? (précisez)

2. La santé dans le service / direction URBANISME - AMÉNAGEMENTS URBAINS - TRANSPORTS

*a. Selon vous, dans la politique qu'il déploie, est ce que le **service/direction urbanisme – aménagements urbains – transports** prend en compte :*

- ☐ Les questions de santé
- ☐ Les inégalités sociales et territoriales de santé
- ☐ Aucune de ces questions
- ☐ Ce service / cette direction n'existe pas
- ☐ Ne sais pas

(plusieurs réponses possibles)

b. Menez-vous des projets communs ou des actions communes avec ce service/direction?

- ○ Oui
- ○ Non
- ○ Ne sais pas

*Si oui, veuillez citer **un exemple** phare d'action commune :*

*c. Siégez-vous (ou votre service) à **un comité ou instance du service/direction urbanisme – aménagements urbains – transports** (type instance d'élaboration du plan local d'urbanisme, programme de renouvellement urbain, schéma de cohérence territoriale, plan de déplacement urbain, zones d'aménagement concerté, ZAC, etc.) ?*

- ○ Oui
- ○ Non
- ○ Ne sais pas

Si oui, le(s)quel(s)?

Si non, pourquoi?

- ○ Ce type de comité/instance n'existe pas dans ma ville
- ○ Ce type de comité n'existe pas mais d'autres modes d'échanges informels existent
- ○ Ce type de comité existe mais je n'y vais pas
- ○ Autre raison (précisez)

Autre raison (précisez) :

Quels autres modes d'échanges informels existent? (précisez)

3. La santé dans le service / direction LOGEMENT - HABITAT

*a. Selon vous, dans la politique qu'il déploie, est ce que le **service/direction logement - habitat** prend en compte :*

- [ ] Les questions de santé
- [ ] Les inégalités sociales et territoriales de santé
- [ ] Aucune de ces questions
- [ ] Ce service / cette direction n'existe pas
- [ ] Ne sais pas

(plusieurs réponses possibles)

b. Menez-vous des projets communs ou des actions communes avec ce service/direction?

- ○ Oui
- ○ Non
- ○ Ne sais pas

*Si oui, veuillez citer **un exemple** phare d'action commune :*

*c. Siégez-vous (ou votre service) à **un comité ou instance du service/direction logement - habitat** (type instance d'élaboration du plan local d'habitat, instance de délivrance d'autorisation individuelle d'urbanisme, permis de construire, etc.) ?*

- ○ Oui
- ○ Non
- ○ Ne sais pas

Si oui, le(s)quel(s)?

Si non, pourquoi?

- ○ Ce type de comité/instance n'existe pas dans ma ville
- ○ Ce type de comité n'existe pas mais d'autres modes d'échanges informels existent
- ○ Ce type de comité existe mais je n'y vais pas
- ○ Autre raison (précisez)

Autre raison (précisez) :

Quels autres modes d'échanges informels existent? (précisez)

4. La santé dans le service / direction ENFANCE - PETITE ENFANCE - JEUNESSE - ÉDUCATION

a. Selon vous, dans la politique qu'il déploie, est ce que le ***service/direction enfance - petite enfance – jeunesse – éducation*** *prend en compte :*

- ☐ Les questions de santé
- ☐ Les inégalités sociales et territoriales de santé
- ☐ Aucune de ces questions
- ☐ Ce service / cette direction n'existe pas
- ☐ Ne sais pas

(plusieurs réponses possibles)

b. Menez-vous des projets communs ou des actions communes avec ce service/direction?

- ○ Oui
- ○ Non
- ○ Ne sais pas

Si oui, veuillez citer ***un exemple*** *phare d'action commune :*

c. Siégez-vous (ou votre service) à ***un comité ou instance du service/direction enfance - petite enfance – jeunesse – éducation*** *(type instance d'élaboration du Plan d'éducation, Plan d'éducation local, Projet/programme/dispositif de réussite éducative, Projet éducatif global, etc.) ?*

- ○ Oui
- ○ Non
- ○ Ne sais pas

Si oui, le(s)quel(s)?

Si non, pourquoi?

- ○ Ce type de comité/instance n'existe pas dans ma ville
- ○ Ce type de comité n'existe pas mais d'autres modes d'échanges informels existent
- ○ Ce type de comité existe mais je n'y vais pas
- ○ Autre raison (précisez)

Autre raison (précisez) :

Quels autres modes d'échanges informels existent? (précisez)

5. La santé dans le service / direction SPORTS - ACTIVITÉS PHYSIQUES - MOBILITÉS ACTIVES

a. Selon vous, dans la politique qu'il déploie, est ce que le ***service/direction sports – activités physiques – mobilités actives*** *prend en compte :*

- ☐ Les questions de santé
- ☐ Les inégalités sociales et territoriales de santé
- ☐ Aucune de ces questions
- ☐ Ce service / cette direction n'existe pas
- ☐ Ne sais pas

(plusieurs réponses possibles)

b. Menez-vous des projets communs ou des actions communes avec ce service/direction?

- ○ Oui
- ○ Non
- ○ Ne sais pas

Si oui, veuillez citer ***un exemple*** *phare d'action commune :*

c. Siégez-vous (ou votre service) à ***un comité ou instance du service/direction sports – activités physiques – mobilités actives*** *(type Plan Sport – Santé, Plan marche, Plan vélo, Plan piéton, etc.) ?*

- ○ Oui
- ○ Non
- ○ Ne sais pas

Si oui, le(s)quel(s)?

Si non, pourquoi?

- ○ Ce type de comité/instance n'existe pas dans ma ville
- ○ Ce type de comité n'existe pas mais d'autres modes d'échanges informels existent
- ○ Ce type de comité existe mais je n'y vais pas
- ○ Autre raison (précisez)

Autre raison (précisez) :

Quels autres modes d'échanges informels existent? (précisez)

6. La santé dans le service / direction RESTAURATION COLLECTIVE - ALIMENTATION

*a. Selon vous, dans la politique qu'il déploie, est ce que le **service/direction restauration collective – alimentation** prend en compte :*

- [ ] Les questions de santé
- [ ] Les inégalités sociales et territoriales de santé
- [ ] Aucune de ces questions
- [ ] Ce service / cette direction n'existe pas
- [ ] Ne sais pas

(plusieurs réponses possibles)

b. Menez-vous des projets communs ou des actions communes avec ce service/direction?

- ○ Oui
- ○ Non
- ○ Ne sais pas

*Si oui, veuillez citer **un exemple** phare d'action commune :*

*c. Siégez-vous (ou votre service) à **un comité ou instance du service/direction restauration collective – alimentation** ?*

- ○ Oui
- ○ Non
- ○ Ne sais pas

Si oui, le(s)quel(s)?

Si non, pourquoi?

- ○ Ce type de comité/instance n'existe pas dans ma ville
- ○ Ce type de comité n'existe pas mais d'autres modes d'échanges informels existent
- ○ Ce type de comité existe mais je n'y vais pas
- ○ Autre raison (précisez)

Autre raison (précisez) :

Quels autres modes d'échanges informels existent? (précisez)

7. La santé dans le service / direction DÉVELOPPEMENT DURABLE

a. Selon vous, dans la politique qu'il déploie, est ce que le ***service/direction développement durable*** *prend en compte :*

- [ ] Les questions de santé
- [ ] Les inégalités sociales et territoriales de santé
- [ ] Aucune de ces questions
- [ ] Ce service / cette direction n'existe pas
- [ ] Ne sais pas

(plusieurs réponses possibles)

b. Menez-vous des projets communs ou des actions communes avec ce service/direction?

- ○ Oui
- ○ Non
- ○ Ne sais pas

Si oui, veuillez citer ***un exemple*** *phare d'action commune :*

c. Siégez-vous (ou votre service) à ***un comité ou instance du service/direction développement durable*** *(type Agenda 21, conseil de la biodiversité, de la nature en ville, etc.) ?*

- ○ Oui
- ○ Non
- ○ Ne sais pas

Si oui, le(s)quel(s)?

Si non, pourquoi?

- ○ Ce type de comité/instance n'existe pas dans ma ville
- ○ Ce type de comité n'existe pas mais d'autres modes d'échanges informels existent
- ○ Ce type de comité existe mais je n'y vais pas
- ○ Autre raison (précisez)

Autre raison (précisez) :

Quels autres modes d'échanges informels existent? (précisez)

8. La santé dans le service / direction ENVIRONNEMENT - ESPACES VERTS

a. Selon vous, dans la politique qu'il déploie, est ce que le ***service/direction environnement – espaces verts*** *prend en compte :*

- [ ] Les questions de santé
- [ ] Les inégalités sociales et territoriales de santé
- [ ] Aucune de ces questions
- [ ] Ce service / cette direction n'existe pas
- [ ] Ne sais pas

(plusieurs réponses possibles)

b. Menez-vous des projets communs ou des actions communes avec ce service/direction?

- Oui
- Non
- Ne sais pas

*Si oui, veuillez citer **un exemple** phare d’action commune :*

*c. Siégez-vous (ou votre service) à **un comité ou instance du service/direction environnement- espaces verts** ?*

- Oui
- Non
- Ne sais pas

Si oui, le(s)quel(s)?

Si non, pourquoi?

- Ce type de comité/instance n’existe pas dans ma ville
- Ce type de comité/instance n’existe pas mais d’autres modes d’échanges informels existent
- Ce type de comité existe mais je n’y vais pas
- Autre raison (précisez)

Autre raison (précisez) :

Quels autres modes d'échanges informels? (précisez)

Partie 3/5 - Collaborations spécifiques avec les services en charge de la nature en ville et des espaces verts

1. La préservation de l'environnement

1. Sur quelles questions liées à la ***préservation de l'environnement au sein des espaces verts*** *collaborez-vous?*

- ☐ Limitation de l'utilisation des pesticides / herbicides / fongicides (dont la politique Zéro phyto)
- ☐ Conservation de la biodiversité
- ☐ Introduction/réintroduction de la nature en ville
- ☐ Réduction du bruit
- ☐ Réduction de la pollution de l'air
- ☐ Limitation des îlots de chaleur en ville
- ☐ Limitation des dommages causés par les inondations
- ☐ Aucun de ces thèmes
- ☐ Ne sais pas
- ☐ Autre sujet concernant l'environnement (précisez)

Autre sujet concernant l'environnement (précisez) : ____________

(plusieurs réponses possibles)

2. Les modes de vie des habitant(e)s

2. Sur quelles questions relatives aux ***modes de vies des habitant(e)s qui sont liés aux espaces verts*** *collaborez-vous?*

- ☐ Promotion de l'activité physique en extérieur (mise en place de parcours de santé, évènement sportif, etc.)
- ☐ Promotion des mobilités douces grâce aux espaces verts : marche, vélo
- ☐ Promotion des espaces de détente et de loisir dans les espaces verts
- ☐ Possibilité de jardinage
- ☐ Aucun de ces thèmes
- ☐ Ne sais pas
- ☐ Autre sujet concernant les modes de vies des habitant(e)s (précisez)

Autre sujet concernant les modes de vies des habitant(e)s (précisez) : ____________

(plusieurs réponses possibles)

3. La préservation de l'équité entre tou(te)s les habitant(e)s

3. Sur quelles questions liées à la ***préservation de l'équité en rapport avec les espaces verts*** *collaborez-vous?*

- ☐ Aménagement d'espaces verts dans les quartiers prioritaires de la ville
- ☐ Facilitation des interactions sociales au sein des espaces verts
- ☐ Accès à un espace vert de loisir libre et gratuit pour tous
- ☐ Accès au un espace cultivable et à de la nourriture saine pour tous (jardin partagé…)
- ☐ Aucun de ces thèmes
- ☐ Ne sais pas
- ☐ Autre sujet concernant la préservation de l'équité entre toutes les personnes vivant dans votre ville (précisez)

Autre sujet concernant la préservation de l'équité entre toutes les personnes vivant dans votre ville (précisez) : ____________

(une ou plusieurs réponses possibles)

4. Autres liens entre la santé et les espaces verts

4. Sur quelles ***questions de santé*** ***liées aux espaces verts*** *collaborez-vous?*

- [ ] Qualité de vie globale des habitant(e)s par les espaces verts
- [ ] Bien-être mental par les espaces verts
- [ ] Prévention des expositions / infections / vecteurs de maladies dans les espaces verts (insectes, nuisibles, tiques, oiseaux… ex : tétanos, maladie de Lyme..)
- [ ] Expositions aux U.V. et prévention solaire dans les espaces verts
- [ ] Limitation des plantes et des espèces allergisantes
- [ ] Sécurité physique des personnes (aménagements spécifiques, barrières de protection, éclairage public, etc.)
- [ ] Prévention des atteintes aux personnes (agressions, violences, etc.)
- [ ] Ne sais pas
- [ ] Autre(s) sujet(s) (précisez)

Autre(s) sujet(s) (précisez) : ______

(plusieurs réponses possibles)

5. Pour l'ensemble de ces questions de cette partie 3...

5. Avec quels autres services de la ville et/ou institutions travaillez-vous en priorité ?

______

Partie 4/5 - Actions du secteur de la santé

1. Les principaux enjeux pour votre ville

*a. Selon vous, quelles sont les **trois actions** phares de votre ville sur les **questions de santé** en 2016/17? (citez ci-dessous, svp)*

*b. Selon vous, quelles sont les **trois actions** phares de votre ville sur les **inégalités sociales et/ou territoriales de santé** en 2016/17? (citez ci-dessous, svp)*

2. Le rôle du service de santé : sensibilisation, plaidoyer

2.1. En interne à la ville

*a. Avez-vous été amené à sensibiliser les **autres services ou directions de la ville**...*

- ☐ aux questions de santé ?
- ☐ aux questions des inégalités sociales de santé?
- ☐ Ne sais pas

(plusieurs réponses possibles)

b. Plutôt à quelle(s) occasion(s) ?

- ☐ Lors du conseil municipal
- ☐ Lors d'un temps décisionnel type réunion de service(s), comité interne
- ☐ Lors d'entretiens informels
- ☐ Lors d'échanges sur un projet commun en cours
- ☐ Lors de journées de formation/sensibilisation pour les agents
- ☐ Autre (précisez)

Autre (précisez) :

(plusieurs choix possibles)

c. Auprès de quel(s) service(s) spécifiques et sur quelle(s) question(s) en particulier?

2.2. En externe

*a. Avez-vous été amené à sensibiliser d'**autres acteurs externes à la ville**...*

- [ ] aux questions de santé ?
- [ ] aux questions des inégalités sociales de santé?
- [ ] Ne sais pas

(plusieurs réponses possibles)

b. Pouvez-vous citer les 3 principaux acteurs que vous avez pu sensibiliser et sur quel(s) thème(s) spécifique(s)?

3. Un comité intersectoriel pour la santé

a. Existe-t-il un comité intersectoriel pour la santé au sein de votre commune ?

- ○ Oui
- ○ Non
- ○ Ne sais pas

Si oui, par qui est-il piloté?

Si oui, y participez-vous ?

- ○ Oui
- ○ Non
- ○ Ne sais pas

A quelle fréquence se réunit-il ?

- ○ Au moins mensuellement
- ○ Au moins trimestriellement
- ○ Au moins annuellement
- ○ Moins d'une fois par an
- ○ Ne sais pas

b. Existe-t-il un/des groupe(s) de travail spécifique(s) dans le cadre du comité intersectoriel pour la santé ?

- ○ Oui
- ○ Non
- ○ Ne sais pas

Si oui, quelles en sont les thématiques phares ?

4. Les collaborations dans le cadre du CLS, PMS, etc.

4. Menez-vous des actions en collaboration avec d’autres services municipaux dans la cadre d’un Contrat local de santé / Plan municipal de santé publique ou autre document cadre de ce type ?

- ☐ Oui, et le(s) CLS/PMS concerne(nt) essentiellement des actions de promotion/prévention/soins
- ☐ Oui, et le(s) CLS/PMS concerne(nt) d'autres thèmes (environnement, habitat,...) et d'autres services sont fortement impliqués
- ☐ Non, nous n’avons pas de document de ce type
- ☐ Ne sais pas

Citez une action phare, svp :

5. Le cas échéant, à quelle(s) autre(s) commission(s) municipale(s) participez-vous?

5. Notez-ici votre réponse, svp :

Partie 5/5 - Personnes en charge des espaces verts

1. Quelles sont au sein de votre commune / intercommunalité, les personnes ressources sur les questions d'espaces verts/nature en ville?

*Nom(s) et prénom(s) du/des **élu(e)**(s) :*

*Nom(s) et prénom(s) du/de la **directeur(trice)** du service :*

2. Coordonnées

*2. Dans le cadre du projet de recherche, nous souhaitons leur faire parvenir **un court questionnaire sur la politique et gestion des espaces verts et de la nature en ville**. Pouvez-vous nous transmettre leur(s) courriel(s)? Vous serez en copie de l'envoi électronique du questionnaire.*

- Oui
- Non

*Adresse courriel de l'/des **élu(e)**(s) :*

*Adresse courriel du/de la **directeur(trice)** du service :*

Remarques

Pour terminer, avez-vous des remarques/commentaires à nous communiquer?
